# Microanastomosis in large vessel size discrepancies: A novel training model

**DOI:** 10.1016/j.jpra.2024.11.002

**Published:** 2024-11-17

**Authors:** Hector Oyonate, Jordi Descarrega, Manuel Fernandez Garrido, Joan Fontdevila

**Affiliations:** Plastic and Reconstructive Surgery Department, Hospital Clinic of Barcelona, Barcelona, Spain

**Keywords:** Microsurgery, Discrepancy, Training model

## Abstract

**Introduction:**

Different vessel diameters may challenge the completion of a high-quality anastomosis in microsurgery. In clinical practice, discrepancies in vessel size are commonly encountered. These variations can range from small to moderate, and microsurgeons typically employ established techniques, such as dilating the smaller vessel or creating an oblique cut in its wall, to address these differences. However, when confronted with larger size discrepancies, there is a lack of consensus on the optimal technique, leading surgeons to rely on their individual experiences. Although various anastomotic techniques have been proposed in recent decades, the absence of a standardised model for practicing large vessel size discrepancy anastomosis has limited comparative research.

Our objective was to develop a new experimental model for practicing large vessel size discrepancy anastomosis using a live rat model.

**Material and Methods:**

Thirty adults Winstar® rats were used to develop a novel training model, the aortofemoral anastomosis, which provides two arterial vessels with a large size discrepancy. Thirty aortofemoral anastomoses were performed using the tapered end-to-end technique by the same operator in an experimental surgery laboratory.

**Results:**

The tapered end-to-end anastomosis technique achieved permeable anastomoses in all 30 models, as assessed using a patency test after 3 h of completion.

**Conclusion:**

The tapered end-to-end anastomotic technique demonstrated satisfactory results in training and clinical practice. However, further research is needed to compare the different anastomotic techniques and determine the optimal approach for large vessel size discrepancies. The aortofemoral anastomosis model stands as a valuable tool for conducting such comparative studies, contributing to the enhancement of microsurgery.

## Introduction

Microsurgery is a relatively new field with a strong experimental foundation. Its inception began in the 1950s and 1960s, when researchers and their teams started exploring various anastomosis techniques under the microscope. This progress was driven by surgical technique and technological advancements, as the existing tools required refinement to function at the microscopic scale. In the late 1960s, the first cases of digit replantation after traumatic amputation were reported. In 1967 and 1968, Chen et al. in Shanghai and Cobbet et al. in England, respectively, reported thumb reconstructions using toe transfers from the foot. In 1972, Buncke and McLean successfully transferred the greater omentum for scalp reconstruction, and in 1973, Daniel and Taylor achieved a successful free transfer of a groin flap.^1^^,^^2^ Since then, plastic and reconstructive surgery was transformed, with microsurgery emerging as a crucial tool for reconstructive surgeons.

During microsurgery, vessel diameter mismatch is a frequent challenge. Small to moderate sized discrepancies are commonly managed using well-known techniques such as dilating the small vessel or creating an oblique cut in the small vessel wall. However, when encountering larger discrepancies, there is no preferred technique, and various solutions exist based on the personal experience of each surgeon. In recent decades, new anastomotic techniques have been described to overcome this issue, including the mouth-fish incision,^3^ ‘small-bite’ technique,^4^ ‘sleeve-anastomosis’,^5^ ‘v-plasty’^6^ and ‘modified Kunlin's’ technique.^7^ Notably, there has been limited research comparing the different anastomotic techniques, partly because there is no established model for practicing large vessel size discrepancy anastomosis.

Our objective was to develop a rat training model for practicing anastomosis in cases of large vessel size discrepancies and establish a basis for comparing different anastomotic techniques in further research.

## Material and Methods

The experimental training model was developed using 30 adult Wistar® rats (weighing 250-300 g). We adhered to all the safety and ethics protocols established by the centre.

**Figure 1:**
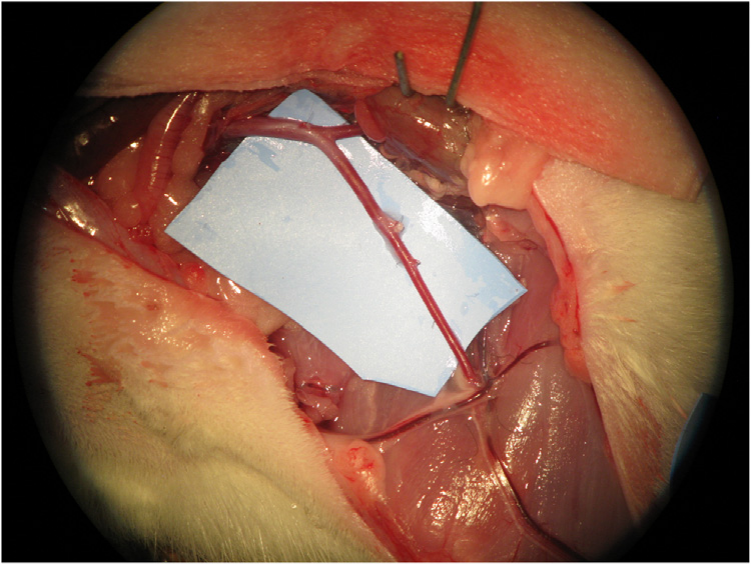
Complete dissection from the aorta to the femoral artery with ligation of all branches.

**Figure 2:**
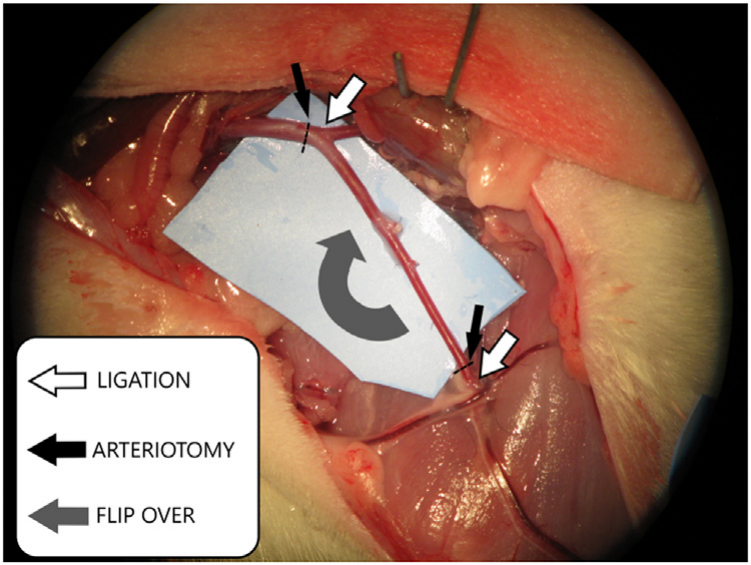
An arteriotomy was performed in the aorta and femoral artery, and the vessels were rotated to match the proximal aorta to the femoral artery.

**Figure 3:**
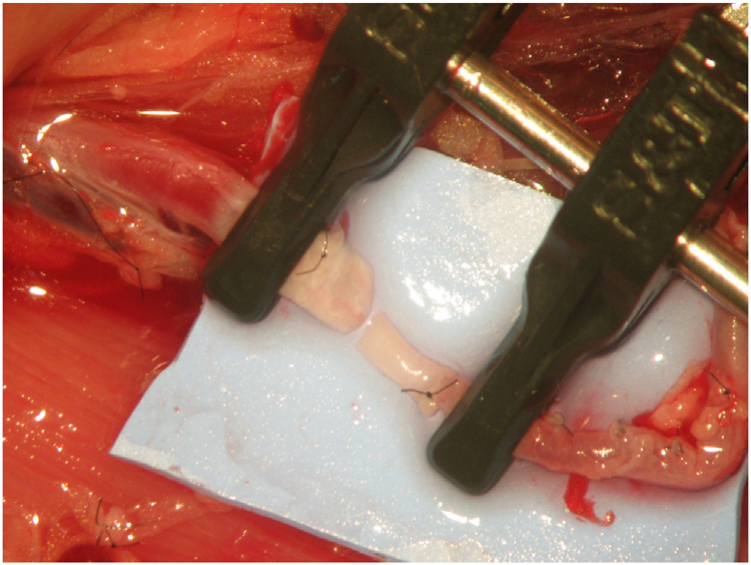
Two arterial vessels with a large diameter discrepancy were obtained.

All surgeries were performed by the same surgeon, under anaesthesia, using local aseptic technique and sterile draping. An adult Wistar® rat was positioned supine with the head towards the surgeon's left hand. An L-shape incision was made on the lower abdomen and groin of the rat, cutting through the full depth of the abdominal wall and inguinal ligament. The abdominal content was retracted to the left side of the animal and protected using a wet gauze to prevent dehydration. The aorta artery, inferior vena cava and femoral vessels were exposed and identified. The arterial vessel was completely dissected from the aorta to the femoral artery by ligating all branches (Figure 1). The aorta was ligated before bifurcation into the common iliac arteries using a 3/0 silk thread. A microvascular clamp was placed on the aorta artery proximal to the ligation. The femoral artery was ligated proximal to the superficial inferior epigastric branch using a 3/0 silk thread. A microvascular clamp was placed on the femoral artery proximal to the ligation. An arteriotomy was performed in the aorta and femoral artery, and the vessels were rotated to match the proximal aorta to the femoral artery (Figure 2). This experimental model provides two arterial vessels with a large diameter discrepancy, allowing for the practice of any anastomotic technique (Figure 3).

Among the available options, we chose to practice the tapered end-to-end anastomotic technique, as described in Flaps and reconstructive surgery by Wei and Mardini.^8^ The microsurgical sutures were performed using 10-0 nylon with a 75 µm needle.

**Figure 4:**
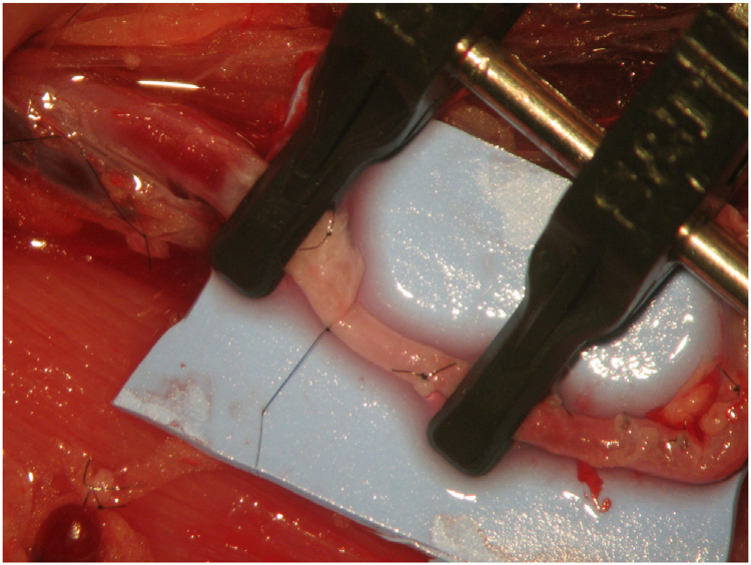
The initial suture was placed at a 0° angle where the two vessels meet.

**Figure 5:**
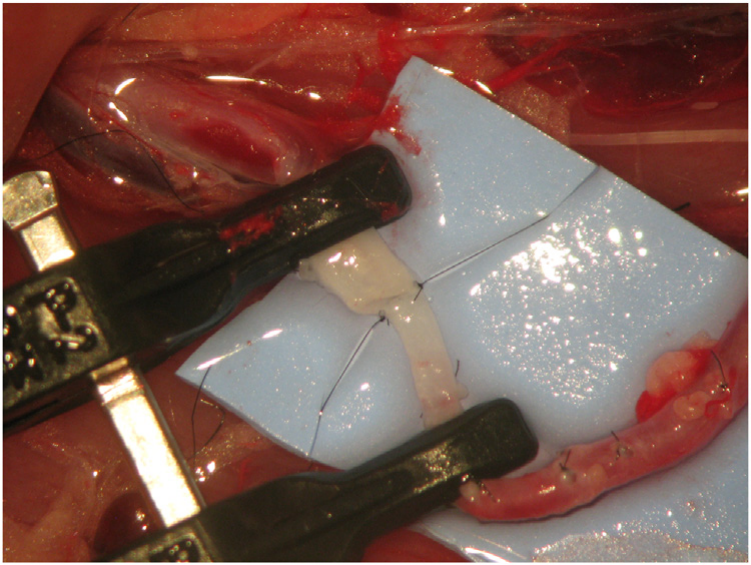
The second stich was inserted at 180° angle from the small vessel taking care to align it with the corresponding point on the larger vessel.

**Figure 6:**
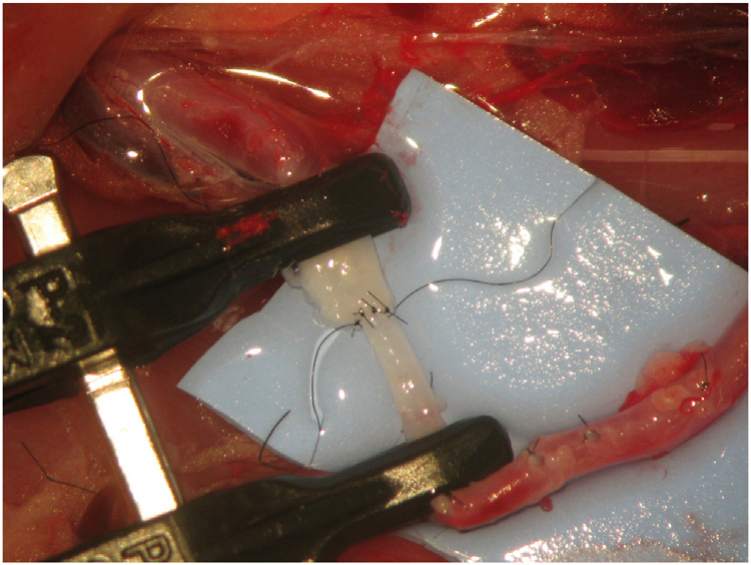
Sutures of the anterior wall were performed.

**Figure 7:**
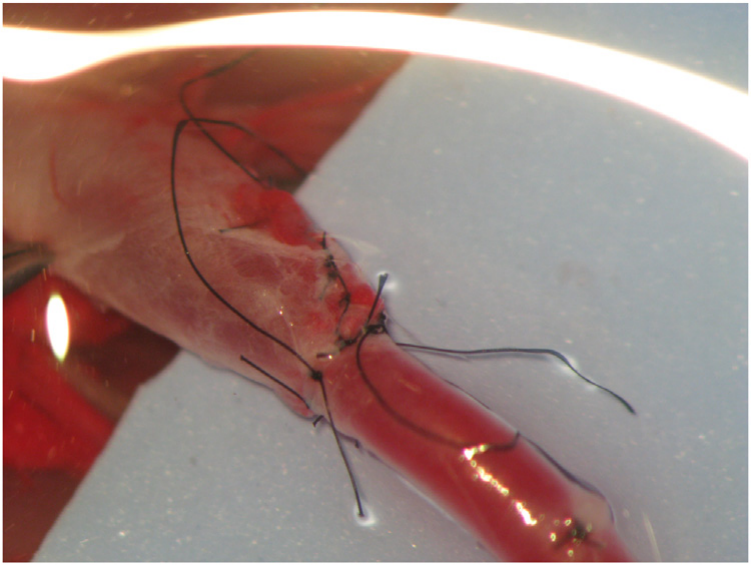
Excision of the redundant portion of the larger vessel wall and suture, creating a tapered end.

**Figure 8:**
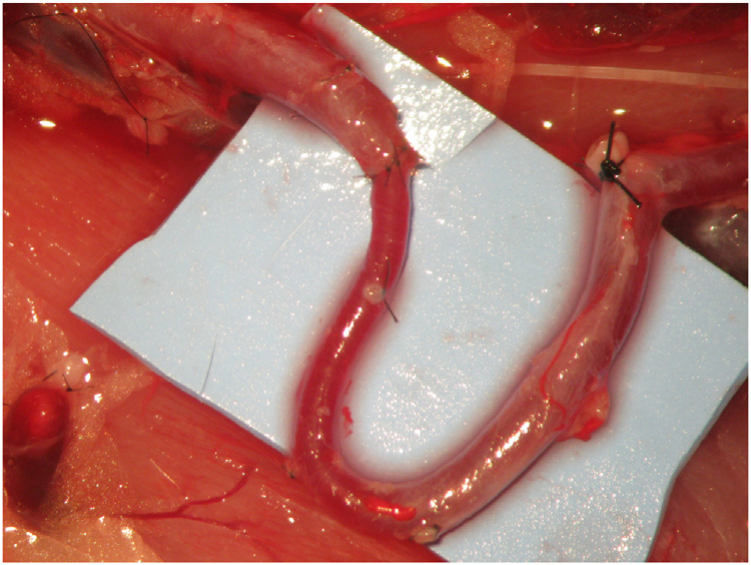
Aortofemoral anastomosis.

The initial suture was placed at a 0° angle where the two vessels meet (Figure 4). The second stich was inserted at 180° angle from the small vessel ensuring that it aligned with the corresponding point on the larger vessel (Figure 5). Subsequently, the anastomotic sutures were placed between these two points on the anterior wall (Figure 6). The clamp was then rotated and the posterior wall was sutured between the 0° and 180° angles. Any redundant portion of the larger vessel wall was excised in a wedge and secured using sutures, resulting in the tapered end of the large vessels (Figure 7, 8).

## Results

The mean time spent on dissecting and preparing the vessels was 95.6±13.57 min. The average time required for performing the tapered end-to-end anastomosis was 31.9±15.94 min. A patent anastomosis was achieved in all 30 subjects, as assessed using a patency test, and remained permeable after 3 h. Adhering to the ethical protocol, all rats were euthanised within 3 to 4 h after the end of the anastomosis.

## Discussion

Preventing large vessel size discrepancies in microsurgery is generally achievable through the careful selection of the flap and recipient vessels. During preoperative planning for free tissue transfer, these considerations—along with factors such as pedicle length and flap thickness—should be considered. However, even with proper surgical planning, unforeseen intraoperative challenges may result in significant vessel size mismatches. Small to moderate discrepancies may be managed using well-known techniques such as dilation of the smaller vessel or creation of an oblique cut in the small vessel wall, but when larger size discrepancies are found, a standardised technique is yet to be established.

Through the decades, different animal models have been developed to facilitate the practice and comparison of different anastomosis techniques in large vessel size discrepancies. In 1989, Gumley et al. used an external jugular vein graft to bridge a femoral artery vessel in a rabbit model and compared 5 different anastomotic techniques; they concluded that end-to-side and tapered end-to-end are the most reliable methods.^9^ This technique added a vein graft to create the discrepancy; furthermore, the rabbit is a more expensive and less available animal model for practicing microsurgery compared to the rat. In 1990, Monsivais described a new animal model in rats, where an inferior vena cava graft was used to bridge the femoral artery to induce a large vessel discrepancy.^10^ In 1994, using this animal model, Ahn et al. compared 3 different techniques, among which the tapered end-to-end anastomosis emerged as the most reliable.^11^ Although the use of a rat animal model enhances reproducibility, the inclusion of a vein graft to induce discrepancy deviates from the surgical experience, where size mismatch would be encountered between two arterial vessels or two venous vessels.

In recent years, several anastomoses have been proposed to address major vessel discrepancies; however, comparative studies using animal models remain limited. More recent research has explored computational models to compare the different anastomotic techniques, and the tapered end-to-end anastomosis has been acknowledged to cause the least flow disturbance. Despite these insights, caution is advised when applying computational model results directly to clinical settings.^12^^,^^13^

In summary, previous animal models for large vessel size discrepancies have predominantly relied on vein grafts to create the size mismatch. Our approach diverges from the typical clinical context, where discrepancies occur more often between vessels of the same type. Our model, which produces a large discrepancy between two arterial vessels, more closely simulates the actual clinical conditions. This new model provides a realistic basis for future studies aimed at comparing various anastomotic techniques under conditions that more closely resemble the operative environment.

## Conclusion

The tapered end-to-end anastomotic technique has shown promising results in training and clinical practice. However, further research is needed to compare various anastomotic techniques and determine the optimal approach for managing large vessel size discrepancies. The aortofemoral anastomosis model stands as a valuable tool for conducting such comparative studies, contributing to the enhancement of microsurgery.
